# OA16.04. Women’s sources of information for CAM use during pregnancy

**DOI:** 10.1186/1472-6882-12-S1-O65

**Published:** 2012-06-12

**Authors:** J Frawley, D Sibbritt, J Adams, A Steel, J Wardle, A Broom, C Lui, V Murthy

**Affiliations:** 1 University of Newcastle, Katoomba, NSW, Australia; 2 University of Technology Sydney, Sydney, Australia; 3 University of Queensland, Brisbane, Australia

## Purpose

It is well known that women of reproductive age are high consumers of complementary and alternative medicine (CAM) with emerging research highlighting the use of high amounts of CAM in pregnancy. Substantial data exists about the information sources women use for utilising complementary medicines and treatments generally, however little is known about the sources of information used in pregnancy. Various authors have raised concerns about aspects of safety in relation to consuming CAM in pregnancy and it is important to elucidate and understand the resources that women are using to gather this information. The objective of our study was to examine women’s information sources when deciding to use CAM in pregnancy.

## Methods

We analysed data from the Australian Longitudinal Study of Women’s Health (ALSWH). The ALSWH is a longitudinal population-based survey which examines the health of a representative sample of over 40,000 Australian women in 3 age groupings. This study has analysed data from a sub-study of 1740 women from the young cohort. These data were supplemented by a review of the literature from the last 10 years.

## Results

Fifty-three percent of women used herbal medicine in pregnancy, whilst only 7.2% visited a herbalist or naturopath; 89.1% consumed vitamins and minerals of which 43.7% self-prescribed these supplements. Women were found to use a variety of sources for information on CAM during pregnancy, namely obstetricians (25.8%) and general practitioners (14.4%), followed by alternative health practitioners (14.3%). Overall, the result for non-professional sources of information (friends, family, internet, magazines etc.) always or sometimes was 61.6% as compared to 61.9% for professional sources.

## Conclusion

Women are using a variety of sources of information about CAM in pregnancy. Many of these are of questionable quality. Health care professionals need to be aware of this and check that women are only consuming CAM products that are safe during pregnancy.

